# Hyperoside Attenuates Bleomycin-Induced Pulmonary Fibrosis Development in Mice

**DOI:** 10.3389/fphar.2020.550955

**Published:** 2020-10-22

**Authors:** Jizhen Huang, Xiang Tong, Li Zhang, Yuan Zhang, Lei Wang, Dongguang Wang, Shijie Zhang, Hong Fan

**Affiliations:** ^1^Department of Respiratory and Critical Care Medicine, West China Hospital/West China School of Medicine, Sichuan University, Chengdu, China; ^2^Department of Ophthalmology, Renmin Hospital of Wuhan University, Wuhan, China

**Keywords:** hyperoside, pulmonary fibrosis, epithelial-mesenchymal transition, oxidative stress, inflammation

## Abstract

Idiopathic pulmonary fibrosis (IPF) is a progressive, lethal, and chronic lung disease. There are no effective drug therapies for IPF. Hyperoside, a flavonoid glycoside, has been proven to have anti-inflammatory, anti-fibrosis, antioxidant, and anti-cancer effects. The aim of this study was to explore the role of hyperoside in bleomycin-induced pulmonary fibrosis development in mice. We established the pulmonary fibrosis model by a single intratracheal aerosol injection of bleomycin. Seven days after the bleomycin treatment, the mice were intraperitoneally administered with hyperoside for 14 days. We found that hyperoside treatment ameliorated fibrotic pathological changes and collagen deposition in the lungs of mice with bleomycin-induced pulmonary fibrosis. Hyperoside treatment also reduced the levels of MDA, TNF-α, and IL-6 and increased the activity of SOD. In addition, hyperoside might inhibit the epithelial-mesenchymal transition (EMT) *via* the AKT/GSK3β pathway. Based on these findings, hyperoside attenuated pulmonary fibrosis development by inhibiting oxidative stress, inflammation, and EMT in the lung tissues of mice with pulmonary ﬁbrosis. Therefore, hyperoside might be a promising candidate drug for the treatment of pulmonary fibrosis.

## Introduction

Idiopathic pulmonary fibrosis (IPF) is a progressive, lethal, and chronic lung disease (Kim et al., 2015). IPF patients have poor long-term survival (Scotton and Chambers, 2007). The incidence of IPF is increasing over time throughout the world (Hutchinson et al., 2015). IPF is characterized by aberrant alveolar epithelial cells, uncontrolled myofibroblast proliferation, and abnormal extracellular matrix (ECM) deposition (Richeldi et al., 2017). EMT plays an important role in many respiratory diseases, including pulmonary fibrosis (Marmai et al., 2011), chronic obstructive pulmonary disease (Xu et al., 2017), and lung cancer (Yin et al., 2020). This transition includes the loss of epithelial markers and the acquisition of mesenchymal markers, which leads to functional changes, including cell migration, invasion, and cell cycle arrest (Willis and Borok, 2007; Lovisa et al., 2015; Yang et al., 2020). Although pirfenidone and nintedanib, which have been approved by the FDA for treating IPF patients, may be able to alleviate the development of IPF, they provide only limited help and entail adverse side effects such as diarrhea and nausea (Canestaro et al., 2016; Galli et al., 2017).

Although the pathogenesis of pulmonary fibrosis is unclear, studies have shown that the transforming growth factor β (TGF-β) plays an important role in the development of pulmonary fibrosis (Yan et al., 2014). TGF-β1 can lead to the phenotypic transition of fibroblasts to myofibroblasts (Wei et al., 2019). TGF-β1 also can induce EMT through smad-dependent pathways or non-smad-dependent pathways, including the phosphoinositide 3 kinase/protein kinase B(PI3K/AKT) pathway and the mitogen-activated protein kinase (MAPK) pathway (Willis and Borok, 2007). The EMT induced by TGF-β1 contributes to the development of pulmonary fibrosis (Willis and Borok, 2007; Zhang C. et al., 2019). The AKT pathway is aberrantly activated in pulmonary fibrosis cellular and animal models (Qu et al., 2019; Ma Z. et al., 2020). The AKT/FOXO3a pathway promotes the proliferation of human pulmonary fibroblasts and the production of collagen (Ma Z. et al., 2020). Other studies have shown that the AKT pathway participates in EMT (Qu et al., 2019). It has been revealed that bleomycin (Blm) can activate the AKT/glycogen synthase kinase 3β (GSK3β) pathway in Blm-induced bronchial epithelial cell injury (Ma et al., 2009). Moreover, the AKT/GSK3β pathway is associated with EMT induced by cigarette smoke extract (Agraval and Yadav, 2019).

Oxidative stress is another significant pathogenesis in the development of pulmonary ﬁbrosis (Cameli et al., 2020). Oxidative stress can contribute to the differentiation of ﬁbroblasts into myoﬁbroblasts (Bai et al., 2018), the apoptosis of alveolar epithelial cells (Liu et al., 2010), and the EMT (Felton et al., 2009). One study found that the level of antioxidants, including superoxide dismutase (SOD) is reduced, and the level of oxidants, including methane dicarboxylic aldehyde (MDA) is increased in the lungs and serum of a rat pulmonary ﬁbrosis model (Bai et al., 2018).

Hyperoside (Hyp) is extracted from Rhododendron brachycarpum G. Don (Ye et al., 2017). Studies have shown that Hyp has numerous biological effects, such as anti-inflammatory (Ye et al., 2017), antioxidant (Ye et al., 2017), anti-fibrosis (Zou et al., 2017), and anti-cancer (Yang et al., 2017) effects. Hyp has been revealed to alleviate allergic airway inﬂammation through the activation of the Nf-E2 related factor 2 (Nrf2) pathway (Ye et al., 2017). Additionally, Hyp was found to have a protective effect on chronic liver fibrosis induced through carbon tetrachloride (Zou et al., 2017). Moreover, Hyp could protect against cardiac remodeling induced by pressure overload through the inhibition of AKT signaling (Wang et al., 2018). In the present study, we explored the protective effects of Hyp on Blm-induced pulmonary fibrosis in a C57BL/6 mice model.

## Materials and Methods

### Materials

Hyp (purity over 98%, B20631) was bought from the Yuanye Biotechnology Co., Ltd. (Shanghai, China). Bleomycin sulfate (S1214) was obtained from Selleck (Shanghai, China). Primary antibodies used in our project were as follows: anti-p-AKT Ser473(Cell Signaling Technology, 4060, dilution 1:2000), anti-AKT(Cell Signaling Technology, 4691, dilution 1:1000), anti-GSK-3β (Cell Signaling Technology, 12456, dilution 1:1000), anti-p-GSK-3β Ser9 (Cell Signaling Technology, 9323, dilution 1:1000), anti-TGF-β1 (Proteintech, 21898-1-AP, dilution 1:1000), anti-SNAIL1 (Proteintech, 13099-1-AP, dilution 1:1000), anti-E-cadherin (Proteintech, 20874-1-AP, dilution 1:1000), anti-a-SMA (Proteintech, 14395-1-AP, dilution 1:1000), anti-vimentin (Cell Signaling Technology, 5741, dilution 1:1000), anti-fibronectin (Proteintech, 15613-1-AP, dilution 1:1000), anti-TWIST1 (Proteintech, 25465-1-AP, dilution 1:1000), anti-N-cadherin (Proteintech, 22018-1-AP, dilution 1:1000), anti-GAPDH (Servicebio Biotechnology Co., Ltd., GB12002, dilution 1:1000), and anti-collagen I (Abcam, ab34710, dilution 1:1000).

### Mouse Models and Treatment

A single intratracheal aerosol injection of Blm (2 mg/kg) dissolved in saline was administered to induce pulmonary fibrosis in C57BL/6 mice (Li et al., 2018). A total of 10 mg of Hyp was dissolved in 20 ul of dimethyl sulfoxide, and then diluted with saline. Hyp was intraperitoneally administered to mice at a dose of 50 mg/kg/d on the basis of a previous study (Jin et al., 2016). The C57BL/6 male mice were procured from the GemPharmatech Co., Ltd. (Jiangsu, China). A total of 32 mice were randomly divided into four groups with eight mice per group: control group, Hyp group, Blm group, Blm plus Hyp group. The control group and Hyp group mice were intratracheally aerosol injected with the same dose of saline. The pulmonary fibrosis mice model was constructed on day 0. Seven days after Blm treatment, the Hyp group and the Blm plus Hyp group mice were intraperitoneally administered with Hyp for 14 days. The control group and Blm group were intraperitoneally administered with a vehicle solution at the same time. Afterwards, the lungs were harvested and weighed on the 21^st^ day. The lung tissues were stored at −80°C and ﬁxed with 10% buﬀered formalin. The lung index was determined as follows: lung index = lung weight (mg)/body weight (g). The animal experimental procedures were approved by the animal ethics committee of West China Hospital, Sichuan University, and consistent with the National Institutes of Health Guide for Care and Use of Laboratory Animals.

### Histopathological Analysis and Immunohistochemical Analysis

After the lung tissues were ﬁxed with 10% buﬀered formalin over 24 h, the lung tissues were embedded in paraffin and cut at 4 µm. Then, the sections underwent hematoxylin-eosin (HE) staining, Masson trichrome staining, and Sirius Red staining. The fibrosis levels were evaluated through the Ashcroft scoring system (Ashcroft et al., 1988). Immunohistochemistry was used to research the level of E-cadherin and a-SMA in the lung tissues. After the sections were dewaxed and rehydrated, they were treated with 3% H_2_O_2_ to inactivate the endogenous peroxidase activity. Then, the sections were blocked through 5% bull serum albumin and incubated with primary antibodies against E-cadherin and a-SMA at a 1:400 dilution. Next, the sections were incubated with secondary antibodies, developed with diaminobenzidine, and stained with hematoxylin.

### Determination of Hydroxyproline, Oxidative Stress, and Inﬂammatory Cytokine in Lung Tissues

The hydroxyproline, SOD (A001-3,Nanjing Jiancheng, Nanjing, China), and MDA (A003-1,Nanjing Jiancheng, Nanjing, China) contents in the mice lung tissues were detected according to the manufacturer’s instructions. The inﬂammatory cytokine levels, including tumor necrosis factor-alpha (TNF-α) (E-EL-M0049c, Elabscience, Wuhan, China) and interleukin 6 (IL-6) (E-EL-M0044c, Elabscience, Wuhan, China), were determined by using an ELISA kit as per the manufacturer’s instructions.

### Real-Time PCR Analysis

Total RNA was isolated from the four groups of homogenized mice lung tissues using the TRIzol reagent (Invitrogen, USA) and then were reverse transcribed into cDNA using a PrimeScript™ RT reagent kit (Takara, Japan) as per the kit’s instructions. Real-time PCR (RT-PCR) was performed with a SYBR Green Kit (Takara, Japan) to determine the mRNA levels of E-cadherin, a-SMA, collagen I, vimentin, twist1, fibronectin, snail1, and N-cadherin, and the 2^-ΔΔCt^ method was used to analyze the data. The primer information of GAPDH, E-cadherin, a-SMA, collagen I, vimentin, twist1, fibronectin, snail1, and N-cadherin are presented as follows. GAPDH-F 5′-CCTCGTCCCGTAGACAAAATG-3′, GAPDH-R 5′-TGAGGTCAATGAAGGGGTCGT-3′;E-cadherin-F 5′-CGACCGGAAGTGACTCGAAAT-3′, E-cadherin-R 5′-TCAGAACCACTGCCCTCGTAAT-3′;a-SMA-F 5′-TCAGGGAGTAATGGTTGGAATG-3′, a-SMA- R 5′-CCAGAGTCCAGCACAATACCAG-3′;collagen I-F 5′-AAGAAGCACGTCTGGTTTGGAG-3′, collagen I-R 5′-GGTCCATGTAGGCTACGCTGTT-3′, vimentin-F 5′-GCAGTATGAAAGCGTGGCTG-3′, vimentin-R 5′-CTCCAGGGACTCGTTAGTGC-3′; twist1-F 5′-CGGCCAGGTACATCGACTTC-3′, twist1-R 5′-TGCAGCTTGCCATCTTGGAG-3′; fibronectin-F 5′-ACACGGTTTCCCATTACGCC-3′, fibronectin-R 5′-GGTCTTCCCATCGTCATAGCAC-3′; snail1-F 5′-AAGCCATTCTCCTGCTCCCA-3′, snail1-R 5′-AGCCAGACTCTTGGTGCTTGTG-3′; N-cadherin -F 5′-CCCTGACTGAGGAGCCTATGAA-3′, N-cadherin –R 5′-GGTTGATAATGAAGATGCCCGTT-3′.

### Western Blot Analysis

Frozen lung tissues were homogenized in RIPA lysis buffer (Beyotime, China). Then, the samples were placed on ice for 10 min and centrifuged for 15 min at 12,000 r/min. After adding 5× protein sample loading buffers (Epizyme, China), the supernatants of the samples were boiled for 10 min. Protein concentrations were detected with a BCA Protein Kit (Beyotime, China). The proteins were separated using 10% SDS-PAGE (Epizyme, China), then transferred onto polyvinylidene difluoride (PVDF) membranes (Millipore, USA) at a constant current of 200 mA. Next, after blocking with 5% non-fat milk for one hour, the membranes were incubated with different primary antibodies against p-AKT, AKT, GSK-3β, p-GSK-3β, TGF-β1, SNAIL1, E-cadherin, vimentin, TWIST1, fibronectin, N-cadherin, a-SMA, collagen I, and GAPDH at 4°C overnight. Subsequently, the membranes were incubated with the appropriate secondary antibodies. The blot was analyzed using the Image J software.

### Statistical Analysis

The experimental data were expressed as the mean ± standard deviation. The differences were analyzed by student’s t tests or one-way ANOVA tests using SPSS 16.0, and the graphs were performed through GraphPad Prism 6.0. A p-value < 0.05 was regarded as statistically significant.

## Results

### Hyp Attenuated Blm-Induced Pulmonary Fibrosis Development in Mice

**Figure 1:**
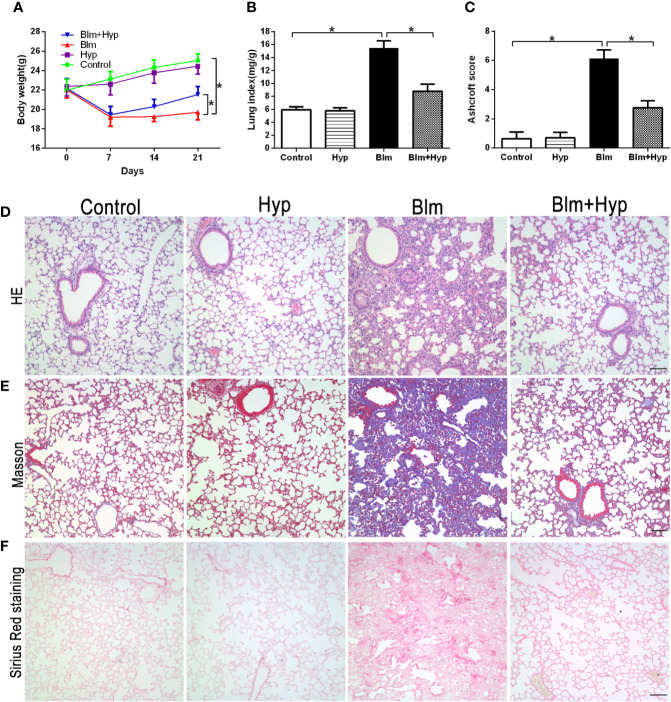
Effect of Hyp on pulmonary fibrosis development induced by Blm in mice. **(A)** Mice body weights were measured (n=8). The pulmonary fibrosis mice model was established on day 0. **(B)** Mice lung index (lung weight/body weight) was calculated on the 21^st^ day (n=8). **(C)** Ashcroft scores for the four groups were based on HE staining (n=4). Representative images of HE staining **(D)**, Masson trichrome staining **(E)**, and Sirius Red staining **(F)** for the four groups (scale bars =50 μm).*P < 0.05.

After a single intratracheal aerosol injection of Blm, the Blm group showed a signiﬁcant body weight loss and lung index increase compared to the control group, and it was dramatically reversed by Hyp intervention (**Figures 1A, B**). Meanwhile, as shown in **Figure 1C**, the Ashcroft scores, which reﬂect the fibrosis levels (Ashcroft et al., 1988), were also markedly increased in the Blm group. Compared with the Blm group, the Blm plus Hyp group had lower Ashcroft scores (**Figure 1C**). Compared to the control group, the Blm treatment contributed to the damaged lung tissue morphological structure, the thickened alveolar wall, and the excessive collagen deposition, as revealed *via* HE staining, Masson trichrome staining, and Sirius Red staining. These effects were dramatically alleviated by Hyp intervention (**Figures 1D–F**).

### Hydroxyproline Content, Oxidative Stress, and Inﬂammatory Cytokine in the Mice Lung Tissues

**Figure 2:**
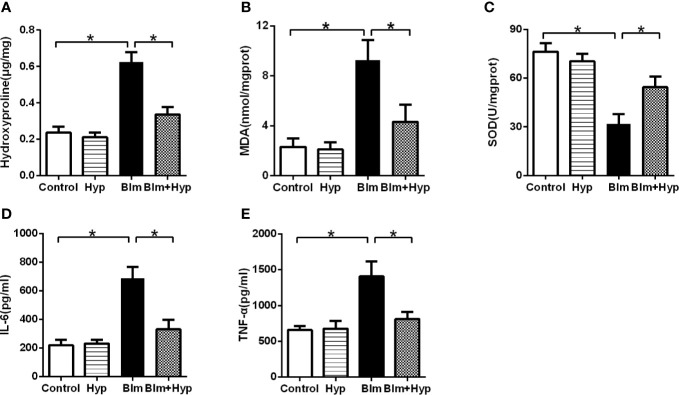
Eﬀect of Hyp on hydroxyproline, oxidative stress, and inﬂammatory cytokine in pulmonary fibrosis mice induced by Blm. The hydroxyproline content **(A)**, the MDA content **(B)**, the SOD activity **(C)**, the IL-6 level **(D)**, and the TNF-α level **(E)** were determined in the mice lung tissues (n=5). *P < 0.05.

As shown in **Figure 2A**, the mouse lung hydroxyproline content (a main ingredient of collagen) was elevated after Blm treatment compared to the control group. However, intervention with Hyp remarkably alleviated the mice’s lung hydroxyproline content. The study found that oxidative stress also plays a significant role in the development of pulmonary ﬁbrosis (Cameli et al., 2020). Hence, to further study the potential mechanisms of the protective effects of Hyp, we measured the oxidative stress in the mice lung tissues. As shown in **Figures 2B, C**, in the mice pulmonary ﬁbrosis model, the activity of SOD, one kind of antioxidant, was dramatically decreased, while the content of MDA, another kind of oxidant, was dramatically increased. However, intervention with Hyp remarkably elevated the activity of SOD and remarkably reduced the content of MDA. In addition, the inﬂammatory cytokines, including IL-6 and TNF-α, were markedly elevated in the mice lung tissues of the pulmonary ﬁbrosis model and were dramatically inhibited by Hyp (**Figures 2D, E**).

### Hyp Inhibited EMT Induced by Blm *In Vivo*

**Figure 3:**
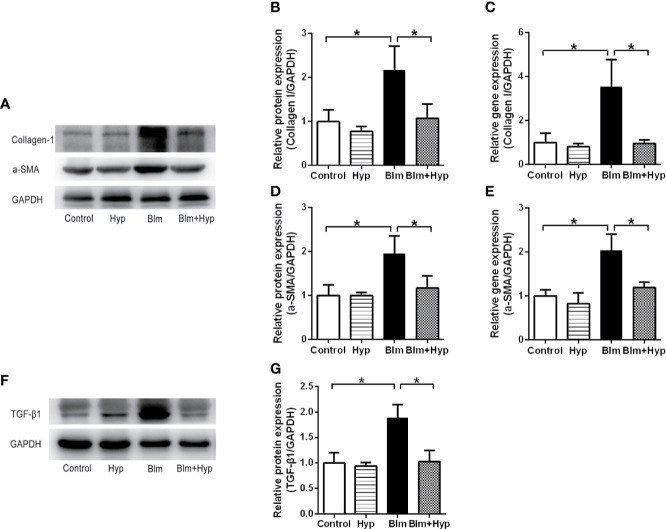
Eﬀect of Hyp on the fibrosis proteins in Blm-induced pulmonary fibrosis in mice. The protein expression of collagen I **(A, B)**, a-SMA **(D)**, TGF-β1 **(F, G)** was determined in each group *via* Western blot analysis (n=3). The gene expression of collagen I **(C)** and a-SMA **(E)** was determined in each group *via* RT-PCR analysis (n=3). *P < 0.05.

**Figure 4:**
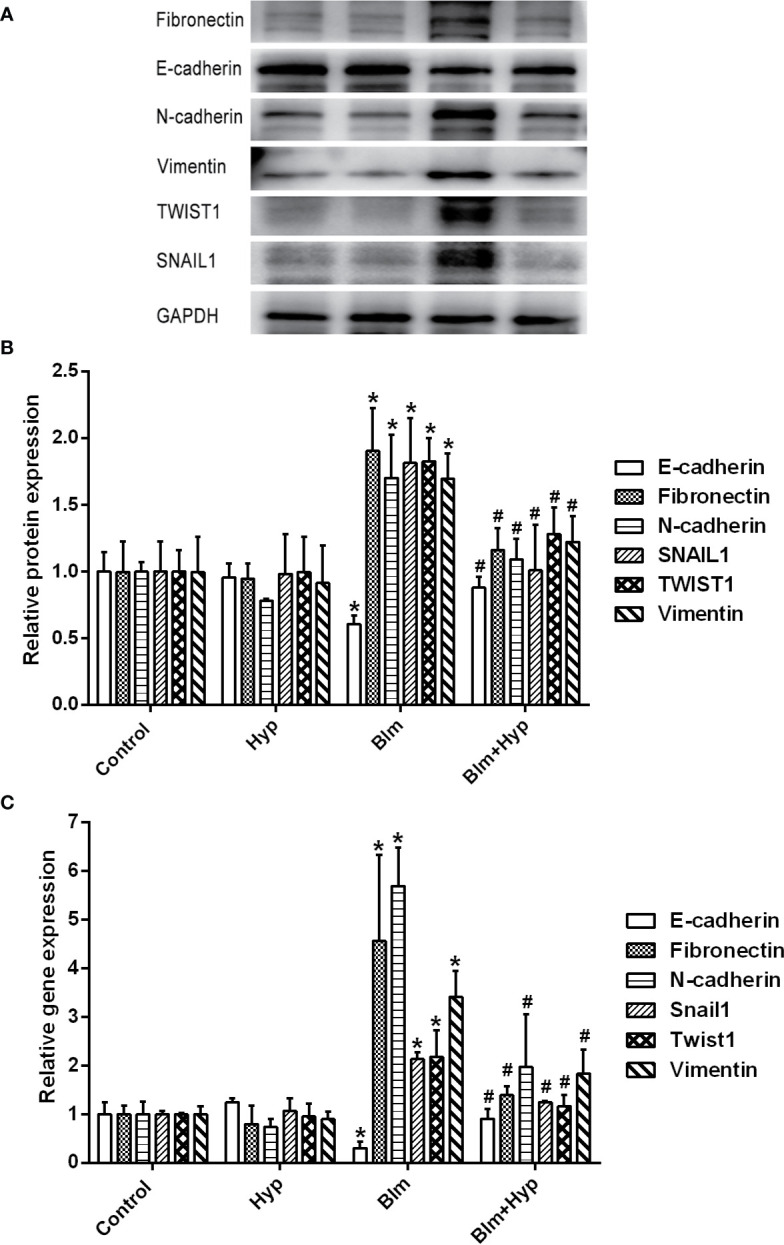
Hyp inhibited EMT induced by Blm *in vivo*. The protein expression of fibronectin, E-cadherin, N-cadherin, vimentin, TWIST1, and SNAIL1 **(A, B)** was determined in each group *via* Western blot analysis (n=3). The gene expression of fibronectin, E-cadherin, N-cadherin, vimentin, twist1 and snail1 **(C)** was determined in each group *via* RT-PCR analysis (n=3). *P < 0.05 compared with the control group; ^#^P < 0.05 compared with the Blm model group.

**Figure 5:**
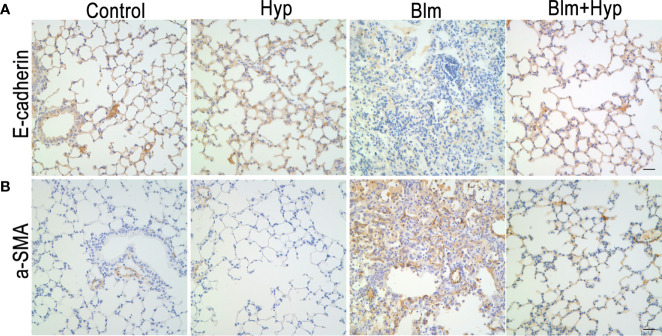
The immunohistochemical analysis of E-cadherin **(A)** and a-SMA **(B)** in mice lung tissues (scale bars =20 μm).

As shown in **Figures 3A–E**, the Western blot and RT-PCR showed that the protein and gene levels of a-SMA and collagen I were up-regulated in the Blm group, and Hyp reduced the expression of a-SMA and collagen I. Hyp also inhibited the expression of TGF-β1 in the mice lung tissues treated with Blm (**Figures 3F, G**). Among the EMT-related markers, the Western blot showed that the protein levels of E-cadherin were down-regulated and the protein levels of fibronectin, N-cadherin, vimentin, TWIST1, and SNAIL1 were up-regulated in the Blm group, and the RT-PCR also showed that the gene levels of E-cadherin were down-regulated and the gene levels of fibronectin, N-cadherin, vimentin, twist1, and snail1 were up-regulated in the Blm group (**Figure 4**). However, compared to the Blm group, the EMT was markedly reversed by the intervention of Hyp. As shown in **Figure 5**, the immunohistochemical staining demonstrated that Blm treatment dramatically reduced the expression of E-cadherin and elevated the expression of a-SMA in mice lungs, and it was also reversed by the intervention of Hyp.

### Hyp Inhibited the AKT/GSK3β Signaling Pathway *In Vivo*

**Figure 6:**
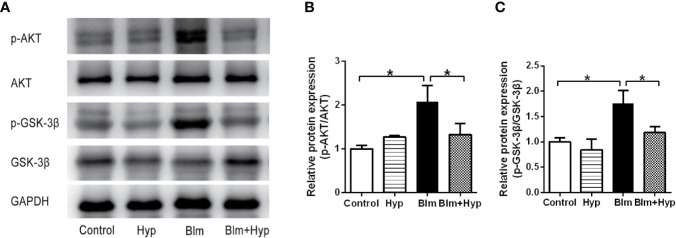
Eﬀect of Hyp on the AKT/GSK-3β signaling pathway in pulmonary fibrosis in mice induced by Blm. The protein expression of p-AKT and p-GSK-3β **(A–C)** was determined in each group *via* Western blot analysis (n=3). *P < 0.05.

To study the potential molecular mechanism of Hyp on pulmonary fibrosis, we detected the effects of Hyp on the AKT signaling pathway. The phosphorylation of AKT and GSK-3β were significantly increased in pulmonary fibrosis mice, and were dramatically inhibited by Hyp (**Figure 6**). GSK-3β is constitutively active and can be inactivated by phosphorylation of Ser9 in an AKT-dependent manner (Grimes and Jope, 2001). Therefore, Hyp intervention blocked activation of AKT and the inactivation of GSK-3β by AKT, and could inhibit the AKT/GSK3β signaling pathway *in vivo*.

## Discussion

Pulmonary fibrosis, the end stage of several diffuse parenchymal lung diseases, is a progressive disease, which leads to eventual death (Wuyts et al., 2013). Although the FDA has approved pirfenidone and nintedanib to treat IPF, none of these markedly decreases patients’ mortality (Canestaro et al., 2016; Galli et al., 2017). Furthermore, few effective drugs can reverse human pulmonary fibrosis and prevent chronic progression to respiratory failure (Li and Kan, 2017). Therefore, it is very meaningful to seek new effective and targeted therapies for pulmonary fibrosis. Recent studies have revealed that the active ingredients from traditional Chinese medicine have anti-fibrotic effects (Li and Kan, 2017). Hyp is an active ingredient extracted from Rhododendron brachycarpum G. Don (Ye et al., 2017). Hyp is known to exert many pharmacological actions, including antioxidant and anti-fibrosis effects (Ye et al., 2017; Zou et al., 2017). Studies have shown that Hyp protects against oxidative damage and cytotoxicity induced by oxalic acid in human kidney-2 cells (Chen et al., 2018). Hyp has also been revealed to prevent heart failure-induced liver fibrosis (Guo et al., 2019). In this study, we revealed that Hyp attenuated Blm-induced pulmonary fibrosis development in mice *via* inhibiting oxidative stress, inflammation, and EMT.

After treatment with Blm, the mouse lung weight dramatically increased due to many factors, including inﬂammatory cell inﬁltration, cell swelling, and capillary congestion, and body weight was decreased (Xin et al., 2019). So, the lung index increased in the pulmonary fibrosis model and reﬂected the degree of pulmonary fibrosis (Xin et al., 2019). As expected, our results showed that the lung index was remarkably increased in the Blm group compared to the control group. After treatment with Hyp, the lung index was decreased.

The main characteristics of pulmonary fibrosis are the activation of myofibroblasts, the deposition of ECM, and the destruction of normal lung structure (Wuyts et al., 2013). Myofibroblasts, transformed from epithelial cells *via* EMT, produce ECM and contribute to the progression of pulmonary fibrosis (Scotton and Chambers, 2007; Zhang L. M. et al., 2019). However, some researchers took the opposite view. Rock et al. thought that epithelial cells were not the origin of myofibroblasts in pulmonary fibrosis (Rock et al., 2011). The origin of myofibroblasts is still controversial. Although it seemed to be a fact that EMT might contribute to pulmonary fibrosis (Willis and Borok, 2007; Zhang C. et al., 2019). We observed Blm-induced pathological changes in mice lung tissues *via* HE staining, Masson trichrome staining, and Sirius Red staining. As expected, the damaged lung tissue’s morphological structure, the thickened alveolar walls, and the excessive collagen deposition were found in the Blm group. Moreover, the Ashcroft scores in the Blm model were significantly higher than in the control group. On the contrary, after treatment with Hyp, these pathological changes were dramatically alleviated. Lung hydroxyproline content, a main ingredient of collagen, was also remarkably reduced by Hyp treatment.

Oxidative stress, an imbalance between oxidants and antioxidants, and inflammation also play important roles in the development and progression of pulmonary fibrosis (Cameli et al., 2020; Otoupalova et al., 2020). One study found that the levels of T-SOD, catalase (CAT), and glutathione (GSH) were significantly decreased, and the expressions of MDA were significantly increased in rat lung and serum after Blm treatment (Bai et al., 2018). The inﬂammatory cytokines, such as TNF-α and IL-6, were also significantly increased after Blm administration (Ma W. H. et al., 2020). Furthermore, the inhibition of oxidative stress and inflammation might ameliorate pulmonary fibrosis in mice models (Ma W. H. et al., 2020). In our investigation, when compared with the control group, the levels of MDA, TNF-α, and IL-6 were dramatically increased and SOD was dramatically reduced in Blm-treated pulmonary fibrosis mice lung tissues. Meanwhile, Hyp markedly inhibited Blm-induced oxidative stress and inflammation in the lung tissues of mice.

TGF-β, one kind of multifunctional cytokine, regulates cell proliferation, cell differentiation, cell apoptosis, and cell migration and favors ECM production (Dennler et al., 2002). The TGF-β signaling pathway is implicated in many diseases, such as cancer, fibrosis, and autoimmune diseases (Dennler et al., 2002). TGF-β1 is an important profibrogenic cytokine in pulmonary fibrosis (Wei et al., 2019). During the progression of pulmonary fibrosis, TGF-β1 induces the differentiation of lung fibroblasts to myofibroblasts and increases the production of collagen (Wei et al., 2019). As expected, our results showed that TGF-β1 was remarkably increased in Blm-treated pulmonary fibrosis mice, and this effect was inhibited by Hyp. Likewise, the levels of a-SMA and collagen I were raised in the Blm group, and Hyp could inhibit the increase of a-SMA and collagen I. Moreover, our study also found that Hyp inhibited the protein and gene levels of fibronectin, N-cadherin, vimentin, TWIST1, and SNAIL1 and increased the protein and gene levels of E-cadherin. However, there was no strong evidence to confirm the contribution of epithelial cells to fibroblasts in our study. Further studies would be needed to confirm the roles of epithelial cells in EMT and pulmonary fibrosis, including lineage tracing studies and cell studies.

The activation of several non-smad-dependent pathways, including the PI3K/AKT pathway and the MAPK pathway, are involved in TGF-β1-induced EMT (Willis and Borok, 2007). The PI3K/AKT pathway plays an important role in cell growth and cell proliferation (Yan et al., 2014). Recent studies have shown that the activation of the AKT pathway plays a considerable role in pulmonary fibrosis (Qu et al., 2019; Ma Z. et al., 2020).

GSK-3β, a serine threonine kinase, is a key downstream target of AKT that is involved in many cellular processes (Grimes and Jope, 2001). GSK-3β is constitutively active and can be inactivated by phosphorylation of Ser9 in an AKT-dependent manner (Grimes and Jope, 2001). One study found that the phosphorylation of AKT was increased in human pulmonary ﬁbroblasts during Blm-induced fibrotic processes (Ma Z. et al., 2020). The phosphorylation of AKT was also increased in radiation-induced pulmonary fibrosis (Qu et al., 2019). Furthermore, during the Blm-induced toxic lung injury, the phosphorylation of AKT at Ser473 and GSK-3β at Ser9 were increased in human bronchial epithelial cells, and the increasing phosphorylation of GSK-3β at Ser9 could inhibit the activity of GSK-3β (Ma et al., 2009). However, the phosphorylation of GSK-3β was decreased after the human bronchial epithelial cells were pretreated with LY294002 (Ma et al., 2009). Cigarette smoke extract induced EMT in airway epithelial cells by the AKT/GSK3β pathway (Agraval and Yadav, 2019). One study found that during the development of Blm-induced pulmonary fibrosis, the up-regulation of SNAIL1 contributed to EMT (Zhang L. M. et al., 2019). Multi-walled carbon nanotubes could prompt the secretion of TGF-β, the activation of AKT, the inhibition of GSK-3β, and the up-regulation of SNAIL1 in human bronchial epithelial cells (Polimeni et al., 2016). This study demonstrated that multi-walled carbon nanotubes could induce EMT by the AKT/GSK-3β/SNAIL1 signaling pathway (Polimeni et al., 2016). Thus, we studied the changes of the AKT/GSK3β signaling pathway in Blm-induced pulmonary fibrosis. In the present study, the p-AKT and p-GSK-3β were significantly elevated in pulmonary fibrosis mice, and these increased expressions were reversed after Hyp treatment. Therefore, Hyp might inhibit EMT *via* the AKT/GSK3β pathway in pulmonary fibrosis. The precise mechanisms between EMT and the AKT/GSK3β pathway need further cell studies.

## Conclusions

**Figure 7:**
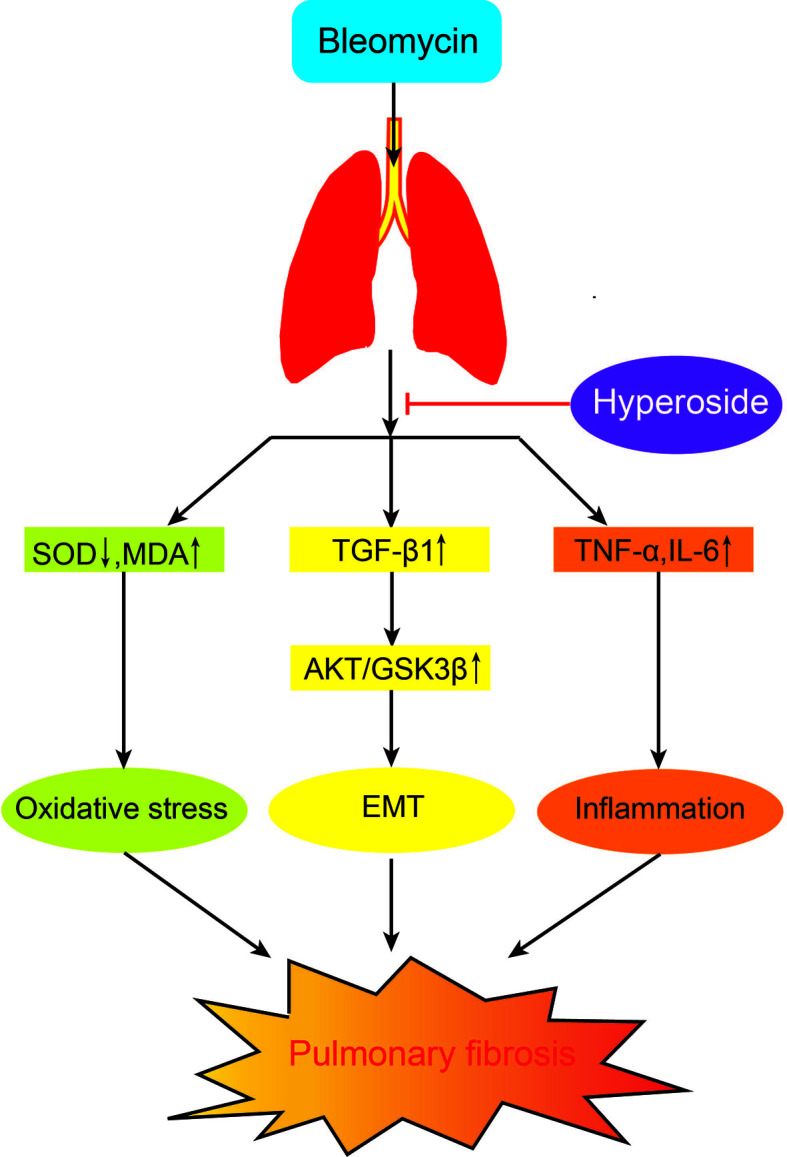
The potential mechanism of Hyp on pulmonary fibrosis mice induced by Blm.

In summary, we revealed that Hyp attenuated pulmonary fibrosis development in mice, and the potential mechanism might be due to inhibiting the Blm-induced inflammation, oxidative stress, and EMT *via* the AKT/GSK3β pathway (**Figure 7**). Hyp might offer a promising candidate drug for the treatment of pulmonary fibrosis.

## Data Availability Statement

The raw data supporting the conclusions of this article will be made available by the authors, without undue reservation.

## Ethics Statement

The animal study was reviewed and approved by the animal ethics committee of West China Hospital, Sichuan University.

## Author Contributions

JH and HF conceived the study. JH, XT,and LZ conducted the experiments. JH wrote the manuscript. JH and YZ conducted the statistical analysis and revised the manuscript. DW and SZ were involved in conducting the experiments. All authors contributed to the article and approved the submitted version.

## Funding

This study was supported by National Key R&D Program of China (2017YFC1309703), China Postdoctoral Science Foundation (2020M673259), and Post-Doctor Research Project, West China Hospital, Sichuan University (2020HXBH013).

## Conflict of Interest

The authors declare that the research was conducted in the absence of any commercial or financial relationships that could be construed as a potential conflict of interest.
